# Direct Position Determination of Wideband Source over Multipath Environment: Combining Taylor Expansion and Subspace Data Fusion in the Cross-Spectrum Domain

**DOI:** 10.3390/s25164967

**Published:** 2025-08-11

**Authors:** Heng Chai, Xinjian Yin, Hao Hu, Xiaofei Zhang

**Affiliations:** 1 College of Electronic Information Engineering, Nanjing University of Aeronautics and Astronautics, Nanjing 211106, China; chaiheng8888@163.com (H.C.); huhao2190@nuaa.edu.cn (H.H.); zhangxiaofei@nuaa.edu.cn (X.Z.); 2 Jiangsu Automation Research Institute, Lianyungang 222000, China

**Keywords:** direct position determination, distributed sensors, multipath environment, Taylor expansion

## Abstract

Position localization of wideband source over multipath environment is addressed in this paper. Traditional methods generally estimate intermediate parameters first and then use these parameters to construct equations for determining the source position. However, the localization accuracy of such methods deteriorates significantly in the presence of multipath effects. In this paper, a direct position determination method combining Taylor expansion and subspace data fusion in the cross-spectrum domain is proposed. The method constructs the data model based on the cross-spectrum of the received signals from arbitrary sensor pairs, effectively avoiding the loss of the available information. Subsequently, forward spatial smoothing is used to address the rank-deficiency problem caused by the multipath effect. Finally, a cost function using subspace data fusion is constructed, and the optimal value is derived via first-order Taylor expansion to compensate for the position estimation bias. The proposed method shows higher localization accuracy compared to state-of-the-art methods. The numerical and experimental results validate the superior localization performance of the proposed algorithm.

## 1. Introduction

Emitter localization techniques, which rely on the distributed passive stations, have significantly contributed to a multitude of fields, including radar and sonar systems, wireless communication, automotive technologies, and signal processing [1,2]. The traditional two-step algorithm [3] is a commonly used position determination method in the early days that contains two independent steps. This method first obtains position-related parameters using the received signals from the observation stations, including the direction of arrival (DOA) [4], time of arrival (TOA) [5], time difference of arrival (TDOA) [6,7], received signal strength (RSS) [8], and frequency difference of arrival (FDOA), etc. Subsequently, the emitter’s position is determined by formulating corresponding equations with the previously obtained parameters. Although the two-step method is widely used, it is still not optimal. The most important reason is that it ignores the correlation between the received signals from the same emitter.

Recently, with the continuous development and improvement of the Direct Positioning Determination (DPD) algorithm [9], this problem has been resolved. In [10], Weiss was the first to systematically introduce this concept and demonstrated its superiority over two-step positioning methods under low SNR conditions. The algorithm employs the Least Square (LS) criterion and Maximum Likelihood (ML) criterion to address the localization problems under unknown noise and known Gaussian noise conditions, respectively. The author in [11] employs a single moving array to collect signals and introduces a novel Subspace Data Fusion (SDF) method, which can be regarded as a generalized form of the MUSIC algorithm [12]. This method significantly improves localization performance and enables the localization of multiple emitters, but requires determining the number of sources in advance. The DPD algorithm described in [13], which leverages the Minimum Variance Distortionless Response (MVDR) approach, offers high-resolution estimation even when dealing with multiple emitters whose numbers are unknown. In [14], the author proposed a DPD algorithm for interference sources in satellite systems based on a propagation operator. This approach reduces the computational complexity of subspace calculations compared with the MUSIC-based methods. In [15], Quantum-behaved Particle Swarm Optimization (QPSO) is applied to determine the optimal array response, thus proposing a Multi-array Data Fusion (MDF)-based technique. In [16], the author proposed a DPD method based on the signal-to-noise ratio (SNR) weighting and the propagation operator to address the issue of varying received SNRs at different observation stations. The author in [17] proposed a closed-form DPD algorithm for linear emitters based on phase alignment and optimal weighted least square (OWLS), referred to as the PAOWLS method.

The aforementioned DPD methods mainly rely on array stations, resulting in significant hardware expenses. In contrast, the DPD method based on TDOA can be implemented using distributed single sensors, yet there is a scarcity of algorithms currently. In [18], the TDOA-based DPD algorithm derived by the maximum likelihood estimator (MLE) is proposed, which provides the Fisher Information Matrix (FIM) in the TDOA scenario, and the author derives the Cramér–Rao Bound (CRB). In [19], the author offers a DPD algorithm for multiple unknown signal emitters based on Hough transform (HT). The author in [20] gives a determinant-based method that uses matrix decomposition and the orthogonal relationship between the noise subspace and the signal manifold to obtain the spectral function. The method delivers superior positioning accuracy for multi-target localization, while the high dimension of the fusion matrix increases computational complexity. Signal time-domain data segmentation is used in [21] to reduce the computational complexity of matrix decomposition. Subsequently, a spectrum function is developed via multiple-frequency function fusion (MFF) to determine the position. In [22], the author introduces a DPD method based on Parallel Factor (PARAFAC), which acquires the time-delay matrix through rapid iteration and then constructs the cost function, providing enhanced performance and reduced complexity. In [23], the authors propose a subspace focusing algorithm for multi-array data fusion, which improves the positioning performance of wideband sources using distributed arrays. In addition, there are still many technologies [24,25,26,27,28,29,30,31,32,33,34,35,36] that have made outstanding contributions to positioning. Most of the aforementioned approaches just consider ideal environments, but only a few algorithms consider localization in multipath environments.

In this paper, a direct position determination algorithm based on TDOA under the multipath environment is proposed. The proposed algorithm jointly employs the cross-spectrum and Taylor compensation, which is proficient in efficiently resolving challenges in multipath environments. The following are the main contributions of this work:

1. We construct the data model based on the cross-spectrum between received signals from any pair of sensors. This method fully extracts the position information from the received signals, thereby avoiding any loss of available information, and can be effectively applied to high-resolution models. Moreover, we further use forward spatial smoothing to address the issue of covariance matrix rank deficiency in multipath environments.
2. We offer a cost function based on forward spatial smooth and subspace data fusion to obtain the initial estimation, and then first-order Taylor expansion is employed to obtain off-grid compensation on the initial estimation, enhancing the localization performance for emitters that do not lie on the search grid.
3. We have evaluated the performance of our algorithm across multiple circumstances using comprehensive simulation studies and actual experiments. The results of these simulations demonstrate our algorithm’s remarkable efficacy and adaptability, underscoring its significant potential for application in multipath environments.

The structure of this paper is as follows: Section 2 describes the model of the received signals. In Section 3, cross-spectrum data model is introduced. Then, Section 4 introduces the proposed algorithm. In Section 5, the proposed algorithm is compared with other algorithms through numerical simulation. Then, the real-world experiment is conducted in Section 6 to verify the viability of the proposed algorithm. Finally, Section 7 concludes the paper.

*Notations*: Bold characters indicate vectors (e.g., a), bold italic characters indicate matrices (e.g., A). ${\left[•\right]}^{T}$, ${\left[•\right]}^{*}$, ${\left[•\right]}^{H}$ and ${\left[•\right]}^{+}$ stand for transpose, conjugation, conjugate transpose and Moore–Penrose inverse, respectively. ${\left[•\right]}_{n}$ denote the *n*-th element of the vector.

## 2. Signal Model

**Figure 1:**
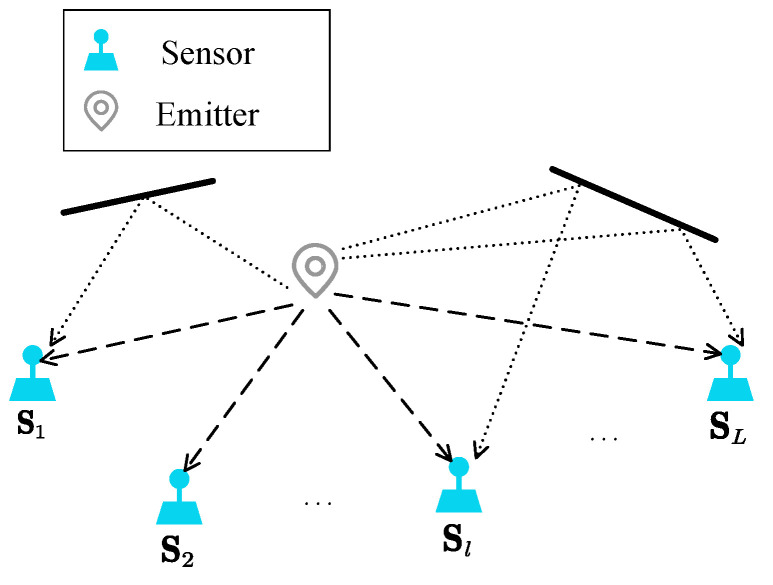
Location scenario in a multipath environment.

Consider that there are *L* observation sensors and the *l*-th sensor located at $S_{l}={\left[X_{l},Y_{l}\right]}^{T}$. We suppose that all sensors are time-synchronized. Suppose that there is an unknown far-field emitter that is confined to a plane with a fixed altitude, and it transmits a wide-band signal. Assuming the unknown emitter is located in $p={\left[x_{p},y_{p}\right]}^{T}$. The localization scenario within a multipath environment is illustrated in Figure 1.

Then, the received signal of the *l*-th sensor can be expressed as

(1) $$x_{l}\left(n\right)=\sum_{m=1}^{M}\lambda_{lm}s\left(n-\tau_{lm}\right)+\sigma_{l}\left(n\right),n=1,2,\ldots ,N_{a},$$

where *M* is the number of multipath components, $N_{a}$ is the number of samples, $\lambda_{lm}$ is the attenuation coefficient caused by the multipath environment, s(n) is the unknown wideband signal transmitted by the emitter, $\sigma_{l}\left(n\right)$ is a white, zero-mean, complex, additive Gaussian noise that is independent of the signal, and $\tau_{lm}$ is the time delay from the emitter to the *l*-th sensor in the *m*-th path.

Considering that the number of sampling points $N_{a}$ is typically large to ensure accurate information capture, to reduce computational complexity and to facilitate the construction of multi-snapshot data, we segment the received data into smaller segments of length $N=N_{a}/K$, where *K* denotes the total number of segments. Then, the *k*-th part of the received signal can be denoted as

(2) $$x_{lk}\left(n_{k}\right)=\sum_{m=1}^{M}\lambda_{lm}s\left(n_{k}-\tau_{lm}\right)+\sigma_{lk}\left(n_{k}\right),n_{k}=1+\left(k-1\right)N,2+\left(k-1\right)N,\ldots ,kN.$$

It should be noted that, although the signal has been segmented, the correlation between these segments remains unchanged.

To extract the time-delay information from the received signal, the cross-correlation function between the signals received by each sensor and the reference signal is typically computed. In this paper, we derive the cross-correlation between any two sensors to fully exploit all the available information. From (2), the *k*-th part’s signal from the *i*-th and *j*-th sensor can be expressed as

(3) $$\left\{\begin{array}{c} x_{ik}\left(n_{k}\right)=\sum_{p=1}^{P}\lambda_{ip}s\left(n_{k}-\tau_{ip}\right)+\sigma_{ik}\left(n_{k}\right)\\ x_{jk}\left(n_{k}\right)=\sum_{q=1}^{Q}\lambda_{jq}s\left(n_{k}-\tau_{jq}\right)+\sigma_{jk}\left(n_{k}\right), \end{array}\right.$$

where *P*, *Q* is the multipath number.

## 3. Pre-Procesing in the Cross-Spectrum Domain

The cross-correlation function can be denoted as

(4) $$R_{ijk}\left(\tau \right)=E\left[x_{ik}\left(n_{k}\right)x_{jk}^{H}\left(n_{k}+\tau \right)\right],$$

where E(·) represents expectation. Considering that signal is independent of the noise, by substituting Equation (3) into Equation (4), it can be rewritten as

(5) $$\begin{array}{cc} R_{ijk}\left(\tau \right)\;\; & =\;\;E\left[\sum_{p=1}^{P}\lambda_{ip}s\left(n_{k}-\tau_{ip}\right)\sum_{q=1}^{Q}\lambda_{jq}^{H}s^{H}\left(n_{k}-\tau_{jq}+\tau \right)\right]\\ \; & =\sum_{p=1}^{P}\sum_{q=1}^{Q}\lambda_{ip}\lambda_{jq}^{H}E\left[s\left(n_{k}-\tau_{ip}\right)s^{H}\left(n_{k}-\tau_{jq}+\tau \right)\right]\\ \; & =\sum_{p=1}^{P}\sum_{q=1}^{Q}\lambda_{ip}\lambda_{jq}^{H}R_{s_{k}}\left(\tau -\left(\tau_{jq}-\tau_{ip}\right)\right)\\ \; & =\sum_{p=1}^{P}\sum_{q=1}^{Q}\lambda_{ip}\lambda_{jq}^{H}R_{s_{k}}\left(\tau -\Delta \tau_{ijpq}\right), \end{array}$$

where $R_{s_{k}}\left(\tau \right)$ is the self-correlation function of $s(n_{k})$ and $\Delta \tau_{ijpq}=\tau_{jq}-\tau_{ip}$. Considering that $R_{ijk}\left(\tau \right)$ and $R_{jik}\left(\tau \right)$ convey identical information, there are L(L−1)/2 unique cross-correlation pairs among the *L* sensors.

By applying discrete Fourier transform (DFT) on $R_{hk}\left(\tau \right)$, the corresponding cross-spectrum is obtained as

(6) $$\begin{array}{cc} G_{ijk}\left(f\right) & =G_{s_{k}}\left(f\right)\sum_{p=1}^{P}\sum_{q=1}^{Q}\lambda_{ip}\lambda_{jq}^{H}e^{-j2\pi f\Delta \tau_{ijpq}}, \end{array}$$

where $G_{s_{k}}\left(f\right)$ is the self-spectrum of $s_{k}\left(n_{k}\right)$.

To utilize the time-delay information for the subsequent processing of the received signal, we assume that the selected reference signal is free of multipath components. In practice, a signal with prior information is typically selected as the reference signal. Then, the reference signal can be written as

(7) $$x_{0k}\left(n_{k}\right)=\lambda_{0}s\left(n_{k}-\tau_{0}\right)+\sigma_{0k}\left(n_{k}\right),$$

where $\lambda_{lm}$ is the attenuation coefficient.

Then, the self-correlation of $x_{0k}\left(n_{k}\right)$ can be denoted as

(8) $$\begin{array}{cc} R_{x_{0k}}\left(\tau \right) & =E[x_{0k}\left(n_{k}\right)x_{0k}^{H}\left(n_{k}+\tau \right)]\\ \; & =E\left[\lambda_{0}s\left(n_{k}-\tau_{0}\right)\lambda_{0}^{H}s^{H}\left(n_{k}-\tau_{0}+\tau \right)\right]\\ \; & +E\left[\sigma_{0k}\left(n_{k}\right)\sigma_{0k}\left(n_{k}+\tau \right)\right]\\ \; & =\lambda_{0}\lambda_{0}^{H}R_{s}\left(\tau \right)+R_{\sigma_{0k}}\left(\tau \right), \end{array}$$

so the self-spectrum can be written as

(9) $$\begin{array}{cc} G_{x_{0k}}\left(f\right) & =\lambda_{0}\lambda_{0}^{H}G_{s_{k}}\left(f\right)+G_{\sigma_{0k}}\left(f\right). \end{array}$$

Then, Equation (6) can be rewritten as

(10) $$\begin{array}{cc} G_{ijk}\left(f\right) & =\left(G_{x_{0k}}\left(f\right)-G_{\sigma_{0k}}\left(f\right)\right)\sum_{p=1}^{P}\sum_{q=1}^{Q}\frac{\lambda_{ip}\lambda_{jq}^{H}}{\lambda_{0}\lambda_{0}^{H}}e^{-j2\pi f\Delta \tau_{ijpq}}. \end{array}$$

Thus, the TDOA information contained in the received signal has been extracted in the form of the cross-spectrum. Equation (10) can be rewritten as

(11) $$\begin{array}{cc} g_{ijk}\left(f\right) & =\frac{G_{ijk}\left(f\right)}{G_{x_{0k}}\left(f\right)}=\sum_{p=1}^{P}\sum_{q=1}^{Q}\mu_{ijpq}e^{-j2\pi f\Delta \tau_{ijpq}}+\gamma_{ijk}\left(f\right) \end{array}$$

(12) $$\begin{array}{cc} \gamma_{ijk}\left(f\right) & =-\frac{G_{\sigma_{0k}}\left(f\right)}{G_{x_{0k}}\left(f\right)}\sum_{p=1}^{P}\sum_{q=1}^{Q}\mu_{ijpq}e^{-j2\pi f\Delta \tau_{ijpq}}, \end{array}$$

where $\mu_{ijpq}=\frac{\lambda_{ip}\lambda_{jq}^{H}}{\lambda_{0}\lambda_{0}^{H}}$, γ(f) can be regarded as the noise term that is independent of $G_{x_{1k}}\left(f\right)$ and follows a Gaussian distribution.

In order to use the high-resolution spectrum estimation method, we would like to sample $g_{ijk}\left(f\right)$ uniformly in the frequency domain to obtain the effective vectors. Let $\omega_{0}$ denote the first chosen frequency point, and we select the valid portion of the cross-spectrum with $N_{f}$ points, then we get

(13) $$G_{ijk}=A_{ij}\mu_{ij}+\gamma_{ijk}\in C^{N_{f}\times 1},$$

where

(14) $$\begin{array}{cc} G_{ijk} & ={\left[\begin{array}{cccc} g_{ijk}\left(f_{0}\right) & g_{ijk}\left(f_{1}\right) & \ldots & g_{ijk}\left(f_{N_{f}-1}\right) \end{array}\right]}^{T}\\ A_{ij} & =\left[\begin{array}{ccccc} a_{ij11}\left(p\right) & \ldots & a_{ijpq}\left(p\right) & \ldots & a_{ijPQ}\left(p\right) \end{array}\right]\\ a_{ijpq}\left(p\right) & ={\left[\begin{array}{cccc} e^{-j2\pi f_{0}\Delta \tau_{ijpq}} & \;e^{-j2\pi f_{1}\Delta \tau_{ijpq}} & \ldots & \;e^{-j2\pi f_{N_{f}-1}\Delta \tau_{ijpq}} \end{array}\right]}^{T}\\ \mu_{ij} & ={\left[\begin{array}{ccccc} \mu_{ij11} & \ldots & \mu_{ijpq} & \ldots & \mu_{ijPQ} \end{array}\right]}^{T}\\ \gamma_{ijk} & ={\left[\begin{array}{cccc} \gamma_{ijk}\left(f_{0}\right) & \gamma_{ijk}\left(f_{1}\right) & \ldots & \gamma_{ijk}\left(f_{N_{f}-1}\right) \end{array}\right]}^{T}, \end{array}$$

where ${\left[•\right]}^{T}$ is transpose, $A_{ij}$ is the signal manifold matrix, and $a_{ijpq}\left(p\right)$ is the signal vector. Combining all data, we can get

(15) $$G_{ij}=\left[\begin{array}{cccc} G_{ij1} & G_{ij2} & \ldots & G_{ijK} \end{array}\right].$$

## 4. The Proposed Algorithm

In this section, based on the cross-spectrum model presented in Section 3, a DPD algorithm is proposed. We call it “DPD-JTAC” as it is based on all cross-spectrum information and Taylor compensation.

### 4.1. Forward Spatial Smoothing Process

Then, the covariance matrix of the ij-th cross-spectrum vector can be denoted as

(16) $${\hat{R}}_{ij}=\frac{1}{K}G_{ij}G_{ij}^{H}=A_{ij}{\hat{R}}_{S,ij}A_{ij}+{\hat{R}}_{W,ij},$$

where ${\hat{R}}_{S,ij}=E\left[\mu_{ij}\mu_{ij}^{H}\right]$ and ${\hat{R}}_{W,ij}=E\left[\gamma_{ijk}\gamma_{ijk}^{H}\right]$.

**Figure 2:**
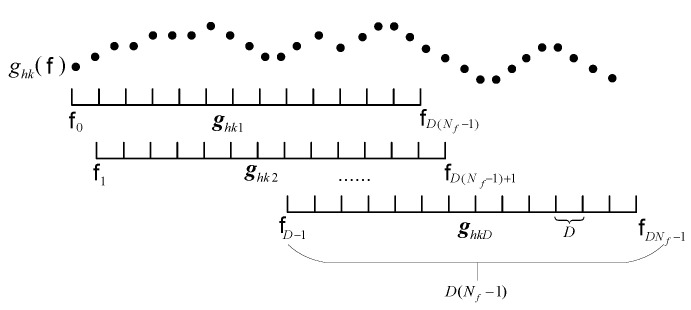
Forward spatial smoothing process.

However, in a multipath environment, $R_{S,ij}$ may not be nonsingular, which means that directly using $R_{ij}$ for high-resolution spectrum estimation is not feasible. Therefore, we subsequently employ the forward spatial smoothing [37], as depicted in Figure 2, to solve this problem. Let $g_{ijkd}\left(d=1,2,\ldots ,D\right)\in C^{N_{f}\times 1}$ denote the *d*-th observation vector of $g_{ijk}\left(f\right)$ and it can be expressed as

(17) $$g_{ijkd}\;=\;{\left[\begin{array}{cccc} g_{ijk}\left(f_{d-1}\right)\;\; & \;\;g_{ijk}\left(f_{d-1+D}\right)]\;\; & \;\;\ldots \;\; & \;\;g_{ijk}\left(f_{d-1+D(N_{f}-1)}\right) \end{array}\right]}^{T},$$

where *D* denotes the sampling interval. Thus, the covariance matrix of the smoothed matrix can be given by

(18) $${\tilde{R}}_{ij}=\frac{1}{K}\sum_{k=1}^{K}G_{ijk}G_{ijk}^{H},$$

where $G_{ijk}=\left[\begin{array}{cccc} g_{ijk1} & g_{ijk2} & \ldots & g_{ijkD} \end{array}\right]$.

### 4.2. Subspace Data Fusion

Then, the eigen-decomposition of ${\tilde{R}}_{ij}$ is performed to obtain the subspace, which can be expressed as

(19) $${\tilde{R}}_{ij}=U_{s}\left(ij\right)\Lambda_{s}\left(ij\right)U_{s}^{H}\left(ij\right)+U_{n}\left(ij\right)\Lambda_{n}\left(ij\right)U_{n}^{H}\left(ij\right),$$

where $\Lambda_{s}\left(ij\right)$ and $\Lambda_{n}\left(ij\right)$ are the diagonal matrices containing the largest eigenvalue and the remaining $N_{f}-1$ eigenvalues, respectively. And $U_{s}\left(ij\right)$ and $U_{n}\left(ij\right)$ represent the signal subspace and noise subspace, respectively, spanned by the corresponding eigenvectors. According to [10], we have the orthogonal relationship in noise subspace and signal vector, thus, we can get

(20) $$a_{ijpq}^{H}\left(p\right)U_{n}\left(ij\right)=0,$$

where 0 denotes the zero vector. Since we only need to determine the true position of the source corresponding to the main path, defining ${\tilde{a}}_{ij}\left(p\right)=a_{ij11}\left(p\right)$, Equation (20) can be rewritten as:

(21) $${\tilde{a}}_{ij}^{H}\left(p\right)U_{n}\left(ij\right)=0.$$

However, due to the influence of noise in the received signal, the above equation can only approximately achieve an orthogonal relationship. Therefore, the cost function at any point $p_{0}={\left[x,y\right]}^{T}$ in the interested area can be denoted as

(22) $$F\left(p_{0}\right)=\;\;\;\sum_{i=1}^{L-1}\sum_{j=i+1}^{L}\frac{1}{{\tilde{a}}_{ij}^{H}\left(p_{0}\right)U_{n}\left(ij\right)U_{ij}^{H}\left(ij\right){\tilde{a}}_{ij}\left(p_{0}\right)},$$

where

(23) $${\tilde{a}}_{ij}\left(p_{0}\right)\;\;=\;\;{\left[\begin{array}{cccc} e^{-j2\pi f_{0}\Delta \tau_{ij11}}\;\; & \;\;e^{-j2\pi f_{D}\Delta \tau_{ij11}}\;\; & \;\;\ldots \;\;\; & \;\;e^{-j2\pi f_{D(N_{f}-1)}\Delta \tau_{ij11}} \end{array}\right]}^{T}$$

(24) $$\Delta \tau_{ij11}=\frac{∥S_{i}-p_{0}∥}{c}-\frac{∥S_{j}-p_{0}∥}{c}.$$

The initial position of the emitter can be determined by searching the spectral peak of the cost function over the meshed region.

### 4.3. Taylor Expansion

The search-based algorithm is generally constrained by the resolution of the search grid, and the position of the emitter may not coincide with the grid point, potentially resulting in biased estimation. To improve the localization accuracy, an off-grid compensation based on the Taylor formula is applied to the initial estimate.

Let $p_{ini}={\left[{\hat{x}}_{ini},{\hat{y}}_{ini}\right]}^{T}$ denote the initial position, in order to use the all cross-spectrum information, we define

(25) $$\begin{array}{c} b\left(p\right)=\left[\begin{array}{c} {\tilde{a}}_{11}\left(p\right)\\ \vdots \\ {\tilde{a}}_{ij}\left(p\right)\\ \vdots \\ {\tilde{a}}_{\left(L-1\right)L}\left(p\right) \end{array}\right]. \end{array}$$

(26) $$\begin{array}{c} U_{n}=\left[\begin{array}{c} U_{n}\left(11\right)\\ \vdots \\ U_{n}\left(ij\right)\\ \vdots \\ U_{n}\left(\left(L-1\right)L\right) \end{array}\right]. \end{array}$$

Due to the orthogonal relationship in noise subspace and signal vector, we have

(27) $$U_{n}^{H}b\left(p\right)=0.$$

Then, perform a first-order Taylor expansion of Equation (25) at $p=p_{ini}$

(28) $$U_{n}^{H}\left(b\left(p_{ini}\right)\;\;+\;\;\frac{\partial b(p_{ini})}{\partial {\hat{x}}_{ini}}\left(x-{\hat{x}}_{ini}\right)+\frac{\partial b(p_{ini})}{\partial {\hat{y}}_{ini}}\left(y-{\hat{y}}_{ini}\right)\right)=0.$$

Defining the deviation term as $\Delta x=x-{\hat{x}}_{ini}$, $\Delta y=y-{\hat{y}}_{ini}$, the least square solution of the deviation term can be denoted as

(29) $$\left[\begin{array}{c} \Delta x\\ \Delta y \end{array}\right]=Re\left[-{\left(U_{n}^{H}\left[\begin{array}{cc} \frac{\partial b(p_{ini})}{{\hat{x}}_{ini}} & \frac{\partial b(p_{ini})}{{\hat{y}}_{ini}} \end{array}\right]\right)}^{+}U_{n}^{H}b\left(p_{ini}\right)\right]$$

According to (25), we can derive that

(30) $$\frac{\partial b(p_{ini})}{\partial {\hat{x}}_{ini}}={\left[\begin{array}{ccccc} \frac{\partial {\tilde{a}}_{11}^{T}\left(p_{ini}\right)}{\partial {\hat{x}}_{ini}}\;\; & \;\;\ldots \;\; & \;\;\frac{\partial {\tilde{a}}_{ij}^{T}\left(p_{ini}\right)}{\partial {\hat{x}}_{ini}}\;\; & \;\;\ldots \;\; & \;\;\frac{\partial {\tilde{a}}_{(L-1)L}^{T}\left(p_{ini}\right)}{\partial {\hat{x}}_{ini}} \end{array}\right]}^{T}.$$

(31) $$\frac{\partial {\tilde{a}}_{ij}\left(p_{ini}\right)}{\partial {\hat{x}}_{ini}}=\frac{\partial \Delta \tau_{ij11}}{\partial {\hat{x}}_{ini}}\left[\begin{array}{c} -j2\pi f_{0}e^{-j2\pi f_{0}\Delta \tau_{ij11}}\\ \vdots \\ -j2\pi f_{D\left(N_{f}-1\right)}e^{-j2\pi f_{D\left(N_{f}-1\right)}\Delta \tau_{ij11}} \end{array}\right].$$

(32) $$\frac{\partial \Delta \tau_{ij11}}{\partial {\hat{x}}_{ini}}=\frac{\left({\left[p_{ini}\right]}_{1}-{\left[S_{i}\right]}_{1}\right)}{∥S_{i}-p_{ini}∥c}-\frac{\left({\left[p_{ini}\right]}_{1}-{\left[S_{j}\right]}_{1}\right)}{∥S_{j}-p_{ini}∥c}.$$

Since the partial derivative of $b(p_{ini})$ with respect to ${\hat{y}}_{ini}$ is of the same form as that with respect to ${\hat{x}}_{ini}$, no further elaboration is provided here. The final estimation of the emitter $\hat{p}={\left[\hat{x},\hat{y}\right]}^{T}$ can be denoted as

(33) $$\left[\begin{array}{c} \hat{x}\\ \hat{y} \end{array}\right]=\left[\begin{array}{c} {\hat{x}}_{ini}\\ {\hat{y}}_{ini} \end{array}\right]+\left[\begin{array}{c} \Delta x\\ \Delta y \end{array}\right].$$

The essential steps of the proposed algorithm are summarized below:

1. Select the reference signal $x_{1}\left(n\right)$ based on the prior information and segment the signals received by each sensor.
2. According to Equation (4), compute the cross-correlation between any two sensors and obtain the cross-spectrum by applying the DFT.
3. Select the effective portion of the cross-spectrum according to Equation (17), and then perform forward spatial smoothing to obtain ${\tilde{R}}_{ij}$ according to Equation (18).
4. Obtain the initial position estimation $p_{ini}$ based on Equations (22)–(24).
5. Obtain the final position estimation $\hat{p}$ based on Equations (25) and (29)–(33).

## 5. Performance Analysis

### 5.1. Complexity Analysis

In this subsection, we contrast the computational complexity of the JTAC algorithm with that of the DPD-MFF [21] algorithm, the DPD-CS [37] algorithm, and the initial estimation of the proposed algorithm (DPD-AC). In this paper, for convenience of comparison, computational complexity is quantified by the number of complex multiplications, which is approximately four times that required for real multiplications. Define $N_{x}$ and $N_{y}$ as the number of grid points for the x-axis and y-axis, respectively. The computational complexity of the cross-correlation is approximately O(N), and that of the cross-spectrum is O(NlogN)). The computational complexity for computing the covariance matrix is $O(N_{f}^{2}D)$, and that for eigenvalue decomposition is $O(N_{f}^{3})$. Subsequently, the computational complexity for computing the cost function is $O\left(L-1\right)LN_{x}N_{y}N_{f}\left(2N_{f}-1\right)/2$, and that of the Taylor compensation is $O(2L\left(L-1\right)\left(4N_{f}^{2}-N_{f}\right)+16N_{f})$. Table 1 summarizes the computational complexities of these four algorithms.

**Figure 3:**
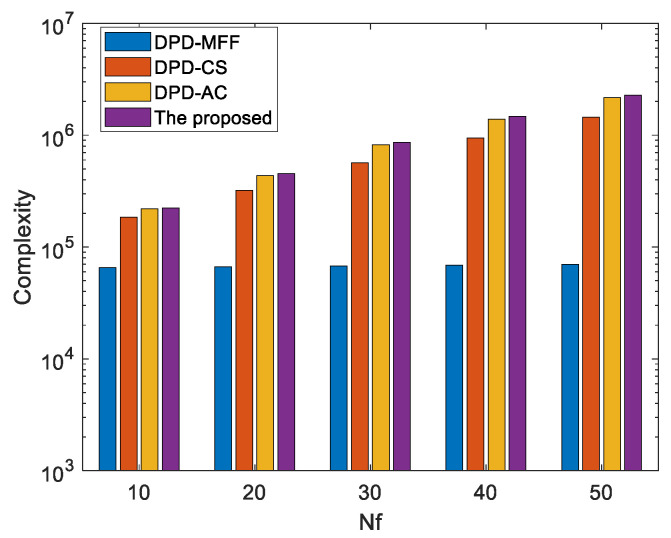
Computational complexity of different algorithm for the different $N_{f}$.

Figure 3 illustrates the complexities of the four algorithms for different $N_{f}$, and the simulation conditions are K=50, L=3, N=256, D=2, $N_{x}=N_{y}=100$, M=3 and $N_{f}$ is changing from 10 to 50. It can be seen that since both the proposed algorithm and the DPD-AC algorithm consider the complete cross-spectrum information, their computational complexity is slightly higher than that of the CS algorithm. However, this increase is minimal while yielding a significant performance improvement. Meanwhile, the proposed algorithm exhibits computational complexity nearly equivalent to that of DPD-AC, while its localization performance is greatly enhanced. On the other hand, although the DPD-MFF algorithm has the lowest computational complexity among the four, its localization performance in multipath environments is extremely poor, as will be clearly demonstrated in the subsequent simulations.

### 5.2. Effectiveness of the Proposed Algorithm

**Figure 4:**
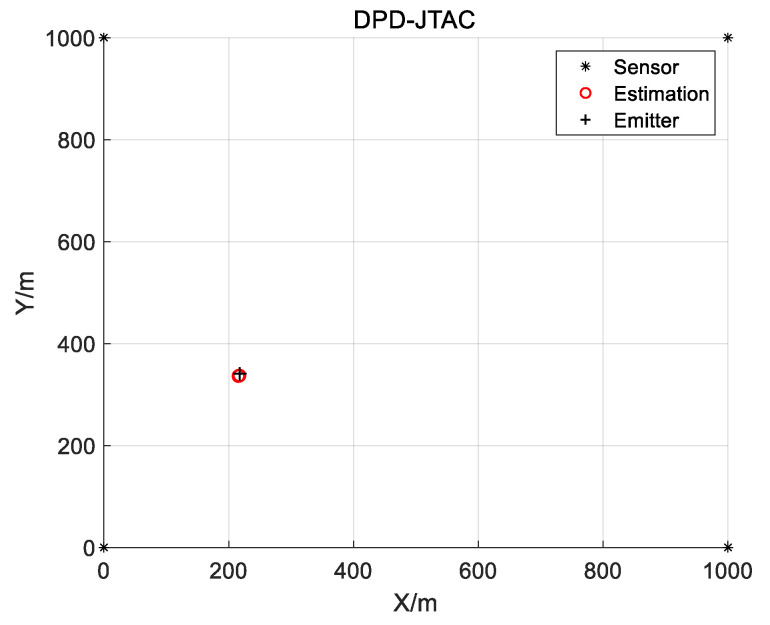
The simulation result.

In this subsection, we use four sensors to localize an emitter to validate the effectiveness of the proposed method. In this simulation, the quadrature amplitude modulation (QAM) signal with a bandwidth of B=32 MHz is used to simulate the emitted signal of the emitter. The simulation parameters are set as $N_{a}=102,400$, N=2048, K=50, $N_{f}=40$, M=3, D=10, the sampling frequency is $f_{s}=125$ MHz and SNR=0 dB. The real position of the emitter is $p={\left[217.74\;m,340.63\;m\right]}^{T}$, the four sensors are located at $S_{1}={\left[0\;m,0\;m\right]}^{T}$, $S_{2}={\left[0\;m,1000\;m\right]}^{T}$, $S_{3}={\left[1000\;m,0\;m\right]}^{T}$, and $S_{4}={\left[1000\;m,1000\;m\right]}^{T}$, respectively. The simulation result with 50 repeated experiments is shown in Figure 4. It is evident that even in the low SNR, the position of the emitter can be determined by the proposed DPD-JTAC algorithm accurately.

### 5.3. Advantage of the Proposed Algorithm

In this subsection, we apply Monte Carlo experiments to estimate the algorithm’s performance to illustrate the advantage of our proposed algorithm. And we use the root mean square error (RMSE) to evaluate the algorithm’s localization accuracy, and it can be denoted as

(34) $$\mathrm{RMSE}=\sqrt{\frac{1}{N_{m}}\sum_{n_{m}=1}^{N_{m}}\left|\right|{\hat{p}}_{n_{m}}-p{\left|\right|}^{2}},$$

where ${\hat{p}}_{n_{m}}$ denotes the position of emitter within the $n_{m}$-th Monte Carlo experiment. In the simulation analysis in this subsection, $N_{m}$ is set to 800.

**Figure 5:**
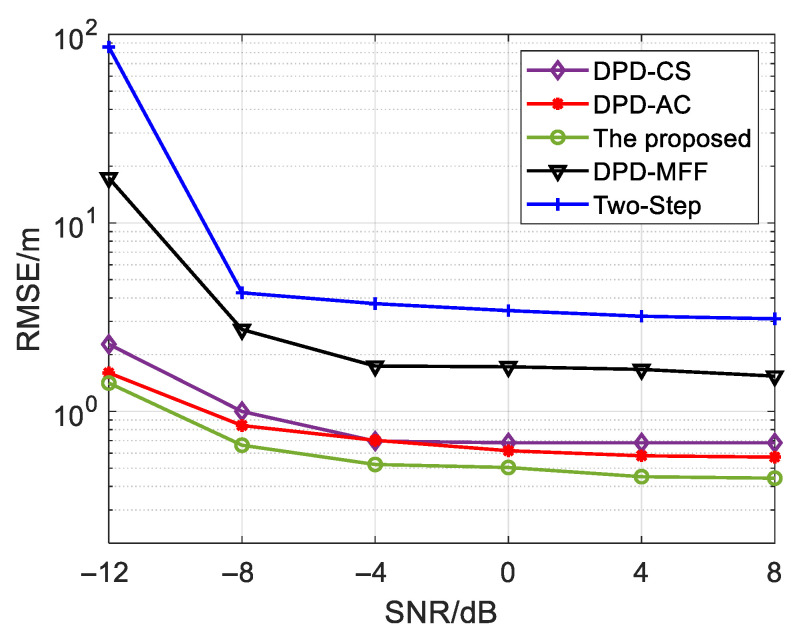
RMSE of various algorithms under different SNRs.

In the first simulation, we compare the performance of the proposed algorithm with the DPD-CS algorithm, the DPD-MFF algorithm, the initial estimation of the proposed algorithm (DPD-AC) and the Two-Step algorithm, under different SNRs by calculating the RMSE using Monte Carlo simulation. The parameters are set the same as Section 5.2 except for the SNR changing from −12 dB to 8 dB and the comparison result is shown in Figure 5. Observably, SNR exerts a significant influence on the proposed algorithm’s performance. The performance of the proposed algorithm is improved and significantly outperforms the other three DPD algorithms as the increases. There are two basic reasons behind this. Firstly, the proposed algorithm employs forward spatial smoothing to eliminate the effects of multipath, thereby achieving performance superior to that of the unprocessed DPD-MFF algorithm. Secondly, the proposed algorithm utilizes the complete cross-spectrum information and applies Taylor compensation on the basis of a coarse estimate, resulting in performance that is undoubtedly superior to that of the DPD-CS algorithm, which uses only L−1 groups of cross-spectrum information, as well as the coarse-estimate DPD-AC algorithm. In addition, the other four algorithms lead to more accurate tracking results compared to the Two-Step algorithm due to the improved likelihood function using the super-resolution cost function.

**Figure 6:**
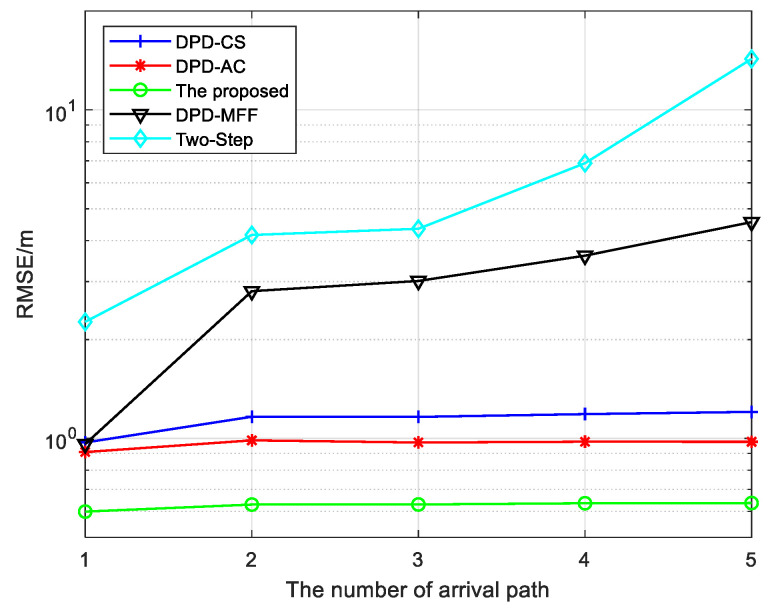
RMSE of various algorithms versus the number of arrival path.

In the second simulation, we analyze the impact of the arrival paths number on the localization accuracy of five algorithms by calculating the RMSE. The other parameters remain the same as the previous simulation, except the number of arrival paths, and SNR is set to 10 dB. The simulation result is shown in Figure 6. The number of arrival paths is increased from 1 to 5. It is clear that as the number of arrival paths increases, the performance of the DPD-MFF and Two-Step algorithms deteriorates drastically, whereas the performance of the other three algorithms remains nearly unchanged. Moreover, the proposed algorithm consistently maintains optimal localization performance, which indicates that it exhibits greater robustness in multipath environment.

Through a comprehensive comparison of Figure 5 and Figure 6, it can be observed that the proposed algorithm effectively adapts to multipath environments, and its performance under such conditions is significantly superior to that of the other algorithms.

## 6. Experiment Result

**Figure 7:**
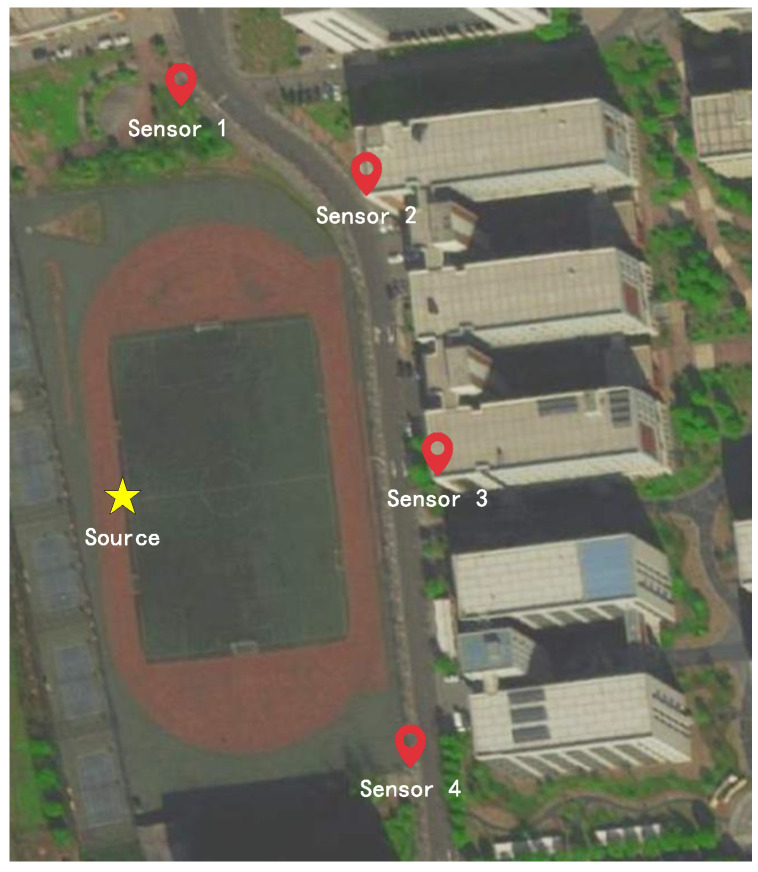
Source and sensing sensor distribution diagram.

**Figure 8:**
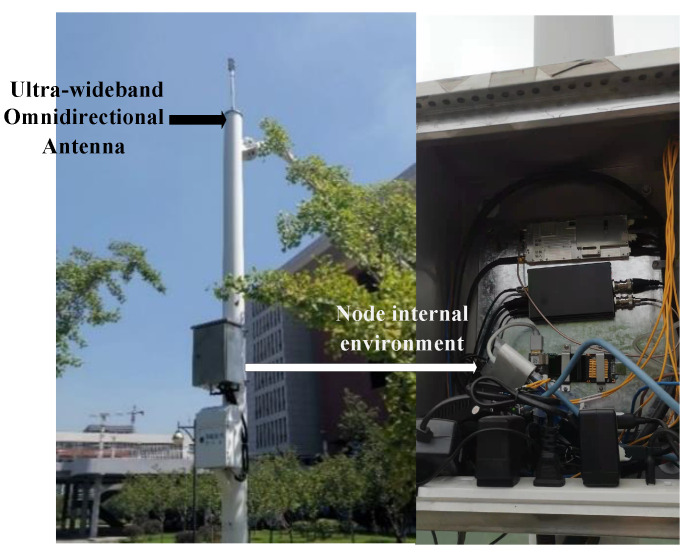
Sensor node and its internal environment.

In this subsection, the real-world experiment is conducted using the sensors on the campus to verify the viability of the proposed algorithm. Figure 7 is the experiment scenario. The source used in this experiment is 1465L-V Signal Generator. The sensor nodes used in the experiment are placed on the rooftops and ground of the campus, consisting of an antenna and a box equipped with node devices. Figure 8 is the schematic diagram of the node and its internal environment.

To verify the effectiveness of the algorithm, we conducted 15 repeated experiments. During each experiment, the source transmitted a 32QAM signal with a bandwidth of 20 MHz and a center frequency of 720 MHz. The sampling frequency used in the experiment is $f_{s}=125$ MHz, the sampling center frequency is $f_{sc}=700$ MHz, and the sampling points during every experiment is 32,508. To facilitate the processing of the received signals, a Cartesian coordinate system is established based on the latitudes and longitudes of the four sensors and the emitter’s location, with Sensor 2 serving as the coordinate origin. The coordinates of four sensors are $S_{1}={\left[-60.1265,32.06\right]}^{T}$, $S_{2}={\left[0,0\right]}^{T}$, $S_{3}={\left[23.5975,-95.4008\right]}^{T}$, $S_{4}={\left[5.3802,-188.353\right]}^{T}$.

**Figure 9:**
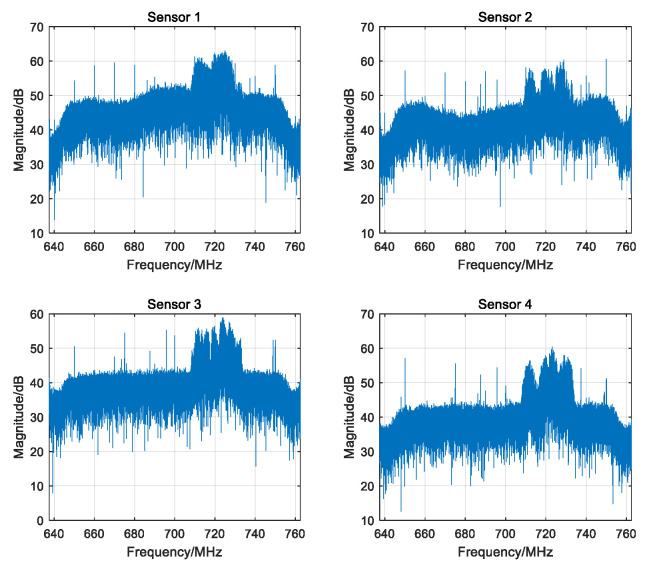
The frequency spectrum of the signals received by the four sensors.

**Figure 10:**
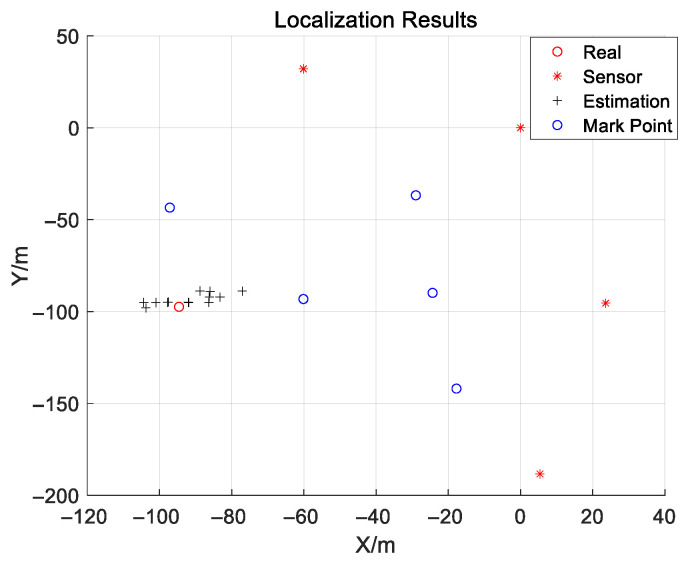
The localization results.

Figure 9 shows the frequency spectrum of the signals received by the four sensors in one experiment, from which the wideband signal can be clearly seen. The localization results of the source with the proposed JTAC algorithm is shown in Figure 10. Clearly, even in a complex and dynamic real electromagnetic environment, the proposed algorithm is also capable of estimating the source’s position.

**Figure 11:**
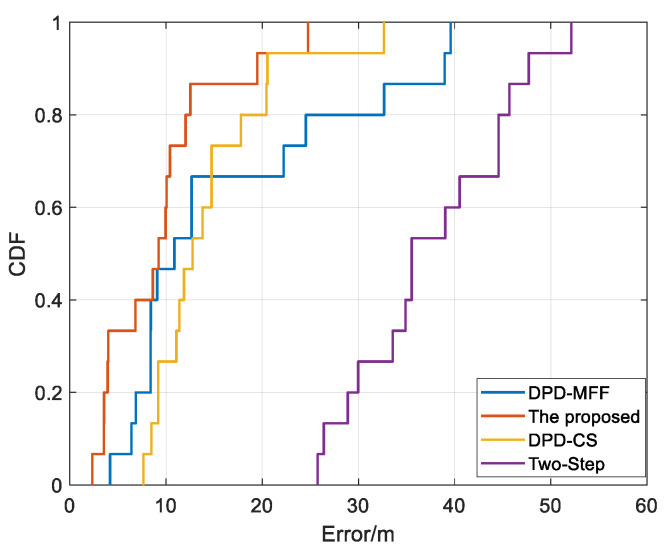
Error CDF curves of different algorithms.

To highlight the advantages of the proposed algorithm, we compared the cumulative distribution function (CDF) curves of localization errors for different algorithms as shown in Figure 11. In comparison to other algorithms, the proposed method aligns most closely with the vertical axis, with almost 90% of the error values falling below 20 m, which demonstrates that the direct positioning algorithm proposed in this paper exhibits lower error compared to other algorithms, indicating its superior localization performance.

## 7. Conclusions

In this paper, a direct position determination method for wideband sources over multipath environments is proposed. Our proposed method introduces a better data model based on the cross-spectrum, which utilizes the received signals from any pair of sensors. Then, we employ forward spatial smoothing to eliminate the multipath effects. Moreover, Taylor expansion is used to obtain the optimal compensation value, enhancing the localization accuracy when the source is not located on the search grid. Finally, the performance analysis, including numerical simulation and real-world experiment, demonstrates the superior localization capability of the proposed method in comparison with existing approaches.

## Figures and Tables

**Table 1:** Complexity of four algorithms.

Algorithm	Computational Complexity
Proposed	$O(KLNlog_{2}N+K\left(L-1\right)L\left(N_{f}^{2}D+N\right)/2+\left(L-1\right)L\left(N_{f}^{3}+N_{x}N_{y}N_{f}\left(2N_{f}-1\right)\right)/2+2L\left(L-1\right)\left(4N_{f}^{2}-N_{f}\right)+16N_{f})$
DPD-AC	$O(KLNlog_{2}N+K\left(L-1\right)L\left(N_{f}^{2}D+N\right)/2+\left(L-1\right)L\left(N_{f}^{3}+N_{x}N_{y}N_{f}\left(2N_{f}-1\right)\right)/2)$
DPD-CS	$O(KLNlog_{2}N+K\left(L-1\right)\left(N_{f}^{2}D+N\right)/2+\left(L-1\right)\left(N_{f}^{3}+N_{x}N_{y}N_{f}\left(2N_{f}-2M+1\right)\right))$
DPD-MFF	$O(LKNlog_{2}N+KN_{f}L^{2}+N_{f}L^{3}+N_{x}N_{y}\left(2N_{f}\left(L-M\right)L^{2}+L^{3}\right))$

## Data Availability

The datasets presented in this article are not readily available because the data are part of an ongoing study. Requests to access the datasets should be directed to xinjianyin@nuaa.edu.cn.

## Abbreviations

The following abbreviations are used in this manuscript:

DPD	Direct Position Determination
DOA	Directory of Arrival
TOA	Time of Arrival
TDOA	Time Difference in Arrival
RSS	Received Signal Strength
FDOA	Frequency Difference in Arrival
LS	Least Square
ML	Maximum Likelihood
SDF	Subspace Data Fusion
MUSIC	Multiple Signal Classification
MVDP	Minimum Variance Distortionless Response
QPSO	Quantum-behaved Particle Swarm Optimization
MDF	Multi-array Data Fusion
SNR	Signal-to-Noise Ratio
OWLS	Optimal Weighted Lest Square
FIM	Fisher Information Matrix
CRB	Cramér–Rao Bound
LFM	Linear Frequency Modulation
STFT	Short-Time Fourier Transform
HT	Hough Transform
MFF	Multiple-frequency Function Fusion
PARAFAC	Parallel Factor
